# Outer-Membrane Permeabilization, LPS Transport Inhibition: Activity, Interactions, and Structures of Thanatin Derived Antimicrobial Peptides

**DOI:** 10.3390/ijms25042122

**Published:** 2024-02-09

**Authors:** Swaleeha Jaan Abdullah, Bernice Tan Siu Yan, Nithya Palanivelu, Vidhya Bharathi Dhanabal, Juan Pablo Bifani, Surajit Bhattacharjya

**Affiliations:** 1 School of Biological Sciences, Nanyang Technological University, Singapore 637551, Singapore; swaleeha002@e.ntu.edu.sg (S.J.A.); nithya.palanivelu@ntu.edu.sg (N.P.);; 2 A*Star Infectious Diseases Labs, 8A Biomedical Grove, Immunos, Singapore 138648, Singapore; 3 Department of Microbiology and Immunology, Yong Loo Lin School of Medicine, National University of Singapore, Singapore 117545, Singapore

**Keywords:** antimicrobial peptide, thanatin, NMR, LPS, LptA, LptA_m_

## Abstract

Currently, viable antibiotics available to mitigate infections caused by drug-resistant Gram-negative bacteria are highly limited. Thanatin, a 21-residue-long insect-derived antimicrobial peptide (AMP), is a promising lead molecule for the potential development of novel antibiotics. Thanatin is extremely potent, particularly against the Enterobacter group of Gram-negative pathogens, e.g., *E. coli* and *K. pneumoniae*. As a mode of action, cationic thanatin efficiently permeabilizes the LPS-outer membrane and binds to the periplasmic protein LptA_m_ to inhibit outer membrane biogenesis. Here, we have utilized N-terminal truncated 16- and 14-residue peptide fragments of thanatin and investigated structure, activity, and selectivity with correlating modes of action. A designed 16-residue peptide containing D-Lys (dk) named VF16 (V1PIIYCNRRT-dk-KCQRF16) demonstrated killing activity in Gram-negative bacteria. The VF16 peptide did not show any detectable toxicity to the HEK 293T cell line and kidney cell line Hep G2. As a mode of action, VF16 interacted with LPS, permeabilizing the outer membrane and binding to LptA_m_ with high affinity. Atomic-resolution structures of VF16 in complex with LPS revealed cationic and aromatic surfaces involved in outer membrane interactions and permeabilization. Further, analyses of an inactive 14-residue native thanatin peptide (IM14: IIYCNRRTGKCQRM) delineated the requirement of the β-sheet structure in activity and target interactions. Taken together, this work would pave the way for the designing of short analogs of thanatin-based antimicrobials.

## 1. Introduction

Infections arising from antibiotic-resistant bacteria are a serious threat to the global healthcare systems. Data from several government agencies and laboratories, including the WHO, have pointed out the global spread of antimicrobial resistance (AMR) that would require immediate interventions [1,2,3]. Based on global AMR data for 2019, a recent study has estimated that 4.9 million deaths are associated with bacterial AMR [4]. Most AMR-related deaths are caused by six pathogens: *E. coli*, *S. aureus*, *K. pneumoniae*, *S. pneumoniae*, *A. baumannii*, and *P. aeruginosa* [4]. Also, *S. aureus*, *K. pneumoniae*, *A. baumannii*, and *P. aeruginosa* are listed by the WHO under the ESKAPE group of bacterial pathogens [5]. Most importantly, infections occur from drug or multi-drug resistance Gram-negative bugs are difficult to treat [6,7,8]. In particular, the emergence of carbapenem, the last resort β-lactam antibiotic, resistant strains of *K. pneumonia*, *A. baumannii,* and *P. aeruginosa* are serious health concerns [6,7,8]. Gram-negative bacteria are known to be intrinsically resistant to many front-line antibiotics, e.g., vancomycin, a consequence of the permeability barrier imposed by the LPS-outer membrane [9,10]. Interests are now growing to develop LPS-outer membrane ‘permeabilizer’ molecules as potentiators or adjuvants for large scaffold antibiotics to kill Gram-negative bacteria [11,12,13].

Host defense AMPs contain a high potential to combat drug or multidrug-resistant bacterial pathogens. AMPs are evolutionary conserved molecules found in all kingdoms of life that are effective in fending off infections as a part of innate immunity [14,15,16,17]. The broad-spectrum activity of AMPs provides vital lead molecules for the next generation of antibiotics [18,19,20]. In particular, the ability of AMPs to kill Gram-negative bacteria can be exploited for the designing of new antibiotics against drug-resistant strains [21,22,23,24]. Notably, multi-drug resistant Gram-negative bacterial infections are now treated with a cyclic cationic peptide polymyxin B and its analogs [25,26]. However, the nephrotoxicity of polymyxin B can significantly restrict its application [27,28]. 

As a part of the mid-gut defense system, a group of insects has been found to express thanatin and thanatin-like highly cationic AMPs [29,30,31]. The 21-residue-long thanatin was isolated from the hemolymph of the bacteria-treated insect *Podisus maculiventris*. A single disulfide bond between residues Cys11 and Cys16 stabilizes the β-hairpin structure of thanatin with a flexible N-terminus region [29]. In animal models of sepsis and infections, thanatin demonstrated lowering of Gram-negative bacterial loads and also neutralization of toxicity of LPS [32,33]. Cationic AMPs are known to utilize membrane disruption mechanisms to exert antibacterial activity [34,35,36]. By contrast, thanatin employs a unique mode of action to kill Gram-negative bacteria. The binding of cationic thanatin with the negatively charged LPS imparts efficient permeabilization of the outer membrane of Gram-negative bacteria [32,37]. This process may facilitate gaining access to AMPs to the periplasmic space of bacteria [38,39,40]. Strikingly, as a part of the mode of action, thanatin demonstrates high-affinity binding with LptA_m_, a truncated functional variant of LptA periplasmic protein of *E. coli*, involved in LPS transport to the outer membrane [41,42,43]. Most Gram-negative bacteria utilize a seven-protein Lpt (LptA to LptG) complex for transport and assembly of newly synthesized LPS at the cell surface of Gram-negative bacteria [44,45,46,47]. As the periplasmic component, LptA essentially serves as a bridge to connect the inner and outer membrane protein complexes of Lpt transport [44,45,46,47]. 

Thanatin-mediated inhibition of LPS transport occurs due to the binding of LptA disrupting LptA and LptC complex [41,42,43]. Thanatin also binds to the periplasmic part of the β-taco domain of LptD to disrupt LPS transport to the outer membrane [48]. The potent activity of thanatin against Gram-negative bacteria, including drug-resistant strains of *E. coli* and *K. pneumoniae*, can be exploited for the further design and development of novel therapeutics [49,50,51]. In this work, we have investigated shorter variants of thanatin in terms of their antibacterial activity, atomic resolution structures, and mode of action. Our study demonstrates correlations of antibacterial activity, LPS-outer membrane permeabilization with β-hairpin structures, and LptA_m_ binding. We believe that the current study might be useful for designing novel thanatin-based AMPs and would essentially enrich the drug discovery pipeline against MDR Gram-negative pathogens. 

## 2. Results

### 2.1. Minimal Inhibitory Concentration (MIC) and Toxicity to HEK293 and Hep2 Cell-Lines

**Figure 1:**
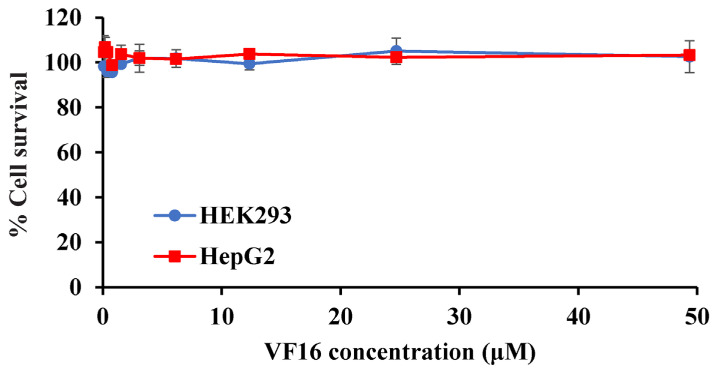
VF16 peptide dose dependence of the survival of the HEK293 and Hep G2 cell lines estimated from MTS assays.

Previous studies have shown that residues at the N-terminus of thanatin (GSKKPVPIIYCNRRTGKCQRM) can be dispensable for antimicrobial activity [29]. By contrast, deletion of residues at the C-terminus of thanatin drastically reduces activity against Gram-negative bacteria [29]. We first examined the antibacterial activity of six and eight residue deletion peptides, namely VM16 (VPIIYCNRRTGKCQRM-amide) and IM14 (IIYCNRRTGKCQRM-amide). VM16 peptide demonstrated growth inhibition against three Gram-negative and also for two Gram-positive bacteria (Table 1). However, antibacterial activity was found to be drastically reduced for IM14 (Table 1). Further, a thanatin analog with M21F substitution demonstrated better antibacterial activity [49]. In addition, residue G11 of thanatin assumes a left-handed helical (α_L_) conformation (ϕ: 66°, ψ: 55°) at the turn, potentially stabilizing the β-hairpin structure [49]. Guided by these observations, we designed an analog peptide of VM16 named VF16 (VPIIYCNRRT-dk-KCQRF-amide), replacing residue Gly with cationic D-Lys and Met to Phe. In particular, the inclusion of the D-Lys would enhance binding with negatively charged LPS-outer membrane, whereas Met to Phe substitution was expected to stabilize aromatic packing interactions within the β-hairpin structure. The designed VF16 showed lower MIC against *E. coli*, whereas similar MIC values were estimated for *K. pneumoniae* and *S. enterica* (Table 1). However, none of the peptides were observed to be active against *A. baumannii*. VF16 peptide was utilized to determine toxicity towards human kidney cell line HEK 293T and liver cell line Hep G2. Figure 1 shows peptide dose dependence of cell survival of HEK 293T and Hep G2 cell lines. As seen, the VF16 peptide is essentially non-toxic as both the cell lines demonstrate a high percentage of survival.

### 2.2. LPS-Outer Membrane Permeabilization and Interactions of the Peptides

**Figure 2:**
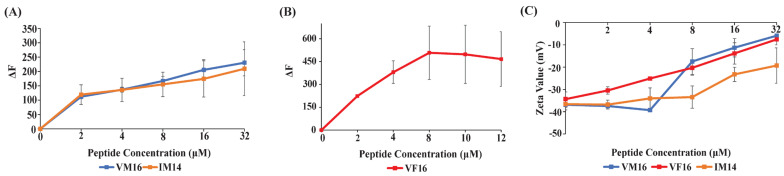
(**A**) Changes of fluorescence intensity (ΔF) of NPN as a function of concentrations of VM16 and IM14 peptides in *E. coli* cell solutions. (**B**) Changes of fluorescence intensity (ΔF) of NPN as a function of concentrations of VF16 peptide in *E. coli* cell solutions. NPN (10 μM) fluorescence studies were conducted in 10 mM sodium phosphate buffer containing 0.5 OD_600_ *E. coli* cell density. The fluorophore was excited at 350 nm, and emission was recorded from 390 to 450 nm at varying concentrations (0 to 16 μM) of VM16, VF16, and IM14 peptides. (**C**) The plot shows changes in bacterial surface charge or ζ potential values as a function of concentrations, 2 to 32 μM, of thanatin peptides, VM16, VF16, and IM14.

**Figure 3:**
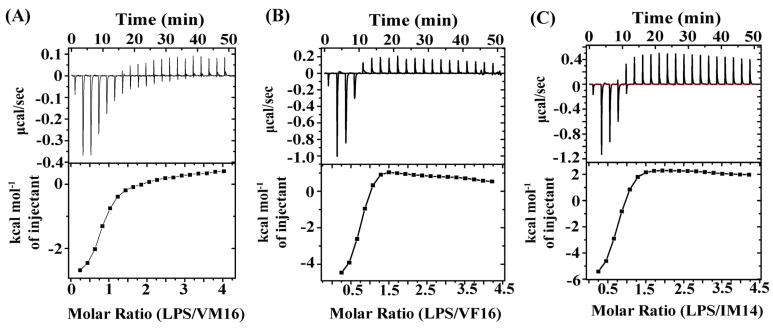
Interactions between LPS with thanatin-derived peptides by ITC, (**A**) VM16, (**B**) VF16, and (**C**) IM14. In an individual experiment, 50 μM of LPS (in a sample cell) was titrated with 2 μL of aliquots of peptides (1 mM, in a syringe) until heat exchange reached saturation. Samples were prepared in 10 mM sodium phosphate buffer, pH 7.0.

We examined the ability of VM16, VF16, and IM14 peptides to permeabilize the LPS-outer membrane, surface charge neutralization, and LPS binding affinity. Uptake of the fluorescent probe 1-N- phenylnaphthylamine (NPN) was utilized to determine the outer-membrane permeabilization of Gram-negative bacteria [52]. Hydrophobic NPN is unable to perturb the LPS-outer membrane and shows a diminished fluorescence emission intensity in a solution of Gram-negative bacteria. However, NPN can readily incorporate into a permeabilized outer membrane, delineating an enhanced fluorescence intensity. In separate NPN assays, aliquots of individual peptides, VM16, VF16, and IM14, were added into buffer solutions containing *E. coli* cells, and NPN and fluorescence emission of the probe were recorded. A dose-dependent increase in fluorescence intensity of NPN was detected for all three peptides, indicating permeabilization of the LPS-outer membrane (Figure 2A,B). However, NPN fluorescence increases for VF16 was estimated to be higher compared to VM16 and IM14 peptides. Interactions of thanatin-derived peptides with bacterial cells were assessed by measuring zeta potential. Gram-negative bacteria are characterized by high negative zeta potential values due to anionic LPS, phospholipids, and carboxylate groups of the cell envelope [53,54]. Bacterial cells can experience a surface charge neutralization effect while binding with cationic AMPs. The process is often found to be correlated with the antibacterial activity of AMPs [53,55]. Titrations of individual peptides, VM16, VF16, and IM14, into *E. coli* cell solutions resulted in the neutralization of negative surface charge (Figure 2C). As seen, VM16 and VF16 peptides afforded a high extent of surface charge neutralization, whereas IM14 peptides appeared to be limited in charge neutralization (Figure 2C). Further, LPS interactions were quantified by use of isothermal titration calorimetry (ITC) experiments whereby individual peptide VM16 (Figure 3A), VF16 (Figure 3B), and IM14 (Figure 3C) were titrated into the sample cell containing LPS in sodium phosphate buffer solution. As evident, binding interactions of thanatin-derived peptides with LPS are exothermic, as seen from negative values in heat exchange in ITC thermograms (Figure 3A–C). Dissociation constant (K_d_) and thermodynamic parameters are provided in Table 2. VF16 and IM14 peptide delineated a tighter binding with LPS compared to VM16. LPS interactions by ITC and analogous biophysical studies are frequently used to determine the mode of action of AMPs [56,57,58].

### 2.3. NMR Studies of the VF16 Peptide in Free Solutions and in LPS

Two-dimensional NMR studies of thanatin peptides were carried out in free solution and in complex with LPS. VM16 peptide appeared to exhibit multiple conformations and overlapping resonances, presumably due to cis-trans conformations of the V-P peptide bond in the peptide. On the other hand, well-resolved NMR spectra were observed for VF16 and IM14 peptides, enabling us to determine their atomic-resolution structures. Analysis of two-dimensional NOESY spectra of VF16 in free solution and tr-NOESY spectra in the presence of LPS revealed diagnostics long-range (>i to i + 4) and medium-range NOE (i to i + 2, i + 3 and i + 4) connectivity involving backbone/backbone, backbone/sidechain and sidechain/sidechain proton resonances. As expected, two-dimensional tr-NOESY spectra of VF16 acquired in the presence of LPS demonstrated many more such NOE contacts, both medium and long-range, for several residues of the peptide.

**Figure 4:**
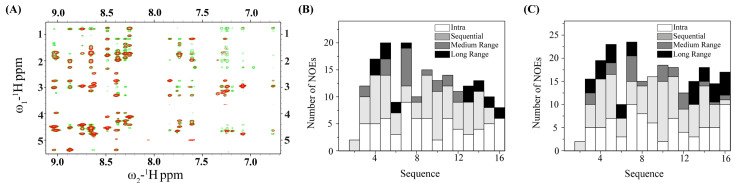
(**A**) Overlay of the selected regions of ^1^H-^1^H two-dimensional NOESY spectra, correlating low-field resonances (6.7–9.1 ppm) along ω_2_ with up-field resonances (0.7–5.4 ppm) along ω_1_ of VF16 peptide in free solution (red contour) and in complex with LPS (green contour). Two-dimensional NOESY spectra of VF16 peptide were acquired in aqueous solutions either in free or in the presence of 20 μM LPS, at 278 K at a peptide concentration of 300 μM. (**B**,**C**) Bar diagrams show types (intra, sequential, medium, and long-range) and number of NOEs observed for VF16 peptide in free solution (**B**) and in the presence of LPS (**C**).

Figure 4A shows an overlay of selected regions of NOESY spectra of VF16, in free solution and in LPS micelle, involving NOEs among the low-field shifted proton resonances along ϖ_2_ dimension with the up-field shifted proton resonances along ϖ_1_ dimension. Table 3 lists representative long-range NOEs of VF16 peptide in free solution and in a complex of LPS micelle. Notably, new long-range NOEs could be detected for residues I3/F16, Y5/F16, Y5/Q14, N7/K12 and C6/C13. The bar diagram summarizes the number and types of NOEs observed for VF16 in free solution (Figure 4B) and in complex with LPS micelle (Figure 4C). As evident, either as free or in LPS micelle, long-range NOEs of VF16 were detected for residues I4, Y5, C6, and N7 at the N-terminus and residues K12, C13, Q14, R15, and F16 at the C-terminus. The intervening residues R8, R9, T10, and dk11 delineated only sequential and medium-range NOE contacts (Figure 4B,C).

### 2.4. Atomic-Resolution Structures of the Active VF16 Peptide in Free Solution and in LPS Micelle

**Figure 5:**
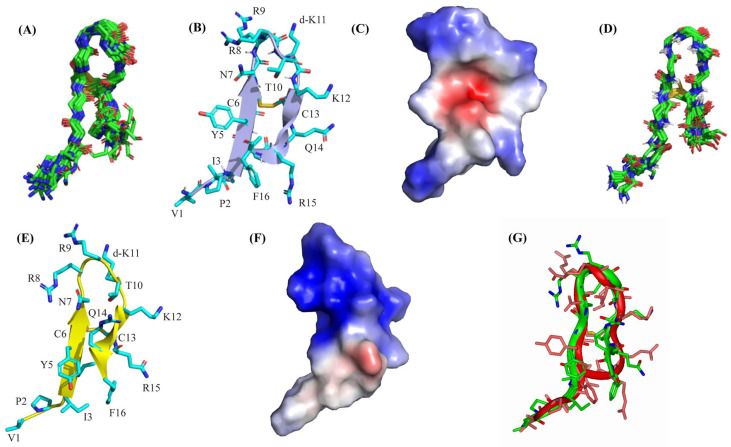
(**A**) Backbone superposition of twenty low-energy structures of VF16 peptide determined in the free state using CYANA. (**B**) Ribbon representation of a selected structure of free VF16 showing β-hairpin topology and dispositions of the sidechains. (**C**) The electrostatic potential surface of VF16 peptide in free solution. Cationic, neutral, and anionic residues are represented as blue, white, and red, respectively. (**D**) Backbone superposition of twenty low-energy structures of VF16 peptide determined in a complex of LPS using CYANA. (**E**) Ribbon representation of a selected structure of VF16 in complex with LPS showing β-hairpin topology and dispositions of the sidechains. (**F**) The electrostatic potential surface of VF16 peptide in complex with LPS. (**G**) Backbone superposition of VF16 peptide in free (red ribbon) and in complex with LPS (green ribbon) showing structural differences of the C- terminal β-strands.

Three-dimensional structures of VF16 in free solution and in complex with LPS were determined by the use of CYANA based on NOE-driven distance and backbone dihedral angle (ϕ, φ) constraints. Structural statistics are summarized in Table 4. Twenty low-energy structures of VF16 in free solution were superposed with estimating backbone (Cα, N, C′) and all heavy atom RMSD values of 1.02 Å and 1.90 Å, respectively (Figure 5A, Table 4). VF16 assumed a canonical β-hairpin conformation supported by two anti-parallel β-strands connected by a loop (Figure 5B). Residues I3-I4-Y5-C6 and residues K12- C13-R14-R15 at the N- and C-termini maintain the two anti-parallel β-strands, whereas five central residues, N7-R8-R9-T10-dk11, connect the two β-stands of the β-hairpin structure of VF16. The electrostatic potential of the VF16 structure in free solution delineated patches of positively charged surfaces (Figure 5C). The 3D structure of VF16 as a complex of LPS was determined using a higher number of long-range NOE constraints compared to free VF16 (Table 4). Figure 5D shows the superposition of twenty low-energy structures of VF16 in an LPS micelle. Backbone (Cα, N, C′) and all heavy atom RMSD values were estimated to be 0.63 Å and 1.14 Å, respectively (Table 4). It may be noted that the RMSD values, in particular for all heavy atoms, are lower in the LPS micelle-bound structure of VF16 (Table 4).

The β-hairpin structure of VF16 is determined to be sustained in a complex of LPS micelle with N-terminal and C-terminal residues and the central loop (Figure 5E). However, the β-hairpin structure of VF16 in the LPS micelle has discernable differences in sidechain/sidechain proximity and packing interactions compared to the free structure. In particular, close packing can be seen among sidechains of residues I3/F16, I4/R15, Y5/N7, Y5/Q14, and aromatic residues Y5/F16 across the two strands and the loop of the β-hairpin structure of VF16 in LPS micelle (Figure 5E). There are potential backbone hydrogen bonds involving residues R8/T10 in the central loop and residues N7/K12 across the antiparallel β-strands. The atomic resolution structure of VF16 in LPS micelle contains a well-defined cationic surface potentially resulting from the organization of the sidechains upon LPS binding (Figure 5F). The superposition of structures of VF16 in LPS and in free solution revealed marked differences with all heavy atoms RMSD of 1.67 Å (Figure 5G). The spatial orientation of the C-terminal β-strand of VF16 structures has rendered significant deviation (Figure 5G).

### 2.5. NMR and 3D Structures of Inactive IM14 Peptide in Free Solutions and in LPS

**Figure 6:**
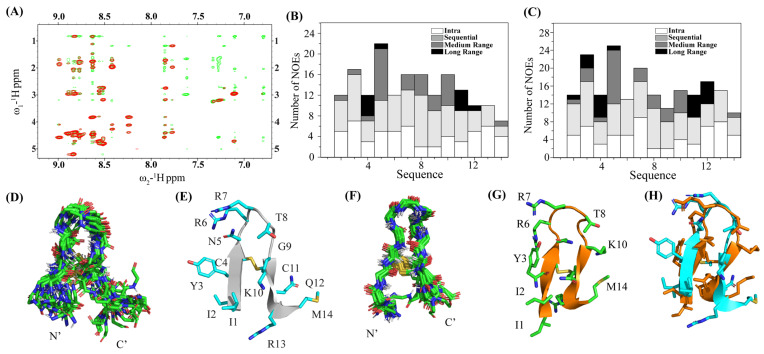
(**A**) Overlay of the selected regions of ^1^H-^1^H two-dimensional NOESY spectra, correlating low-field resonances (6.7–9.1 ppm) along ω2 with up-field resonances (0.7–5.4 ppm) along ω1 of IM14 peptide in free solution (red contour) and in complex with LPS (green contour). Two-dimensional NOESY spectra of the peptide were acquired in aqueous solutions either in free or in the presence of 20 μM LPS, at 278 K at a peptide concentration of 300 μM. (**B**,**C**) Bar diagrams show types (intra, sequential, medium, and long-range) and the number of NOEs observed for IM14 peptide in free solution (**B**) and in the presence of LPS (**C**). (**D**) Backbone superposition of twenty low-energy structures of IM14 peptide obtained in free solution using CYANA. (**E**) Ribbon representation of a selected structure of IM14 in the absence of LPS showing dispositions of the sidechains and distorted β-strand at the C-terminus of the β-hairpin structure. (**F**) Backbone superposition of twenty low-energy structures of IM14 peptide obtained in complex with LPS using CYANA. (**G**) Ribbon representation of a selected structure of IM14 in complex with LPS showing backbone topology and dispositions of the sidechains. (**H**) Backbone superposition of the structures of IM14 peptide in LPS (orange ribbon) and in free solution (cyan ribbon) showing structural differences.

Two-dimensional ^1^H-^1^H NOESY spectra of IM14 peptide in free solution and in complex with LPS show significant differences in terms of NOEs observed (Figure 6A). Overall, many more NOEs could be detected for the IM14 peptide in a complex of LPS compared to a free peptide (Figure 6B,C). Notably, the IM14 peptide delineated only nine long-range NOEs, whereas as many as sixteen long-range NOEs could be found in complexes with LPS (Table 5). Residues I2 and Y3 of IM14, in free solution, did not show diagnostic long-range NOEs of β-hairpin conformation with the C-terminal residues. Figure 6C shows superpositions of twenty low-energy structures of IM14 in the absence of LPS with estimated RMSDs of 1.30 Å and 2.37 Å, for backbone and all heavy atoms, respectively (Figure 6D, Table 4). A rather close superposition can be seen for the peptide in the complex with LPS, with reduced RMSD values of 0.79 Å and 1.87 Å for the backbone and all heavy atoms, respectively (Figure 6E, Table 4). Despite the presence of a disulfide bond, the β-hairpin structure of IM14 in free solution appears to contain a significant distortion at the C-terminus (Figure 6F). At the same time, the two anti-parallel β-strands of IM14 were observed to be more closely juxtaposed in complex with LPS (Figure 4G). Overlay of the free and LPS-bound structures of IM14 peptide demarcates significant differences in spatial orientation of the β-strands and loop residues (Figure 6F).

### 2.6. Binding Interactions of VF16 and IM14 Peptides with LPS Transport Periplasmic Proteins

**Figure 7:**
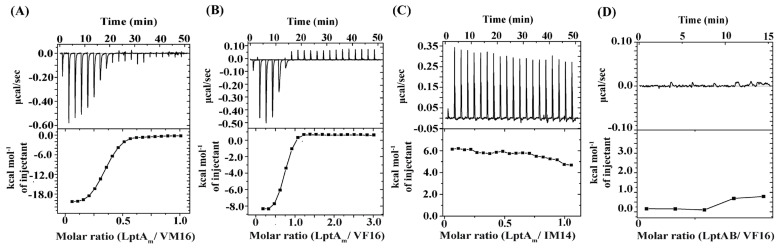
ITC studies of interactions between *E. coli* LptA_m_ (**A**–**C**) with thanatin-derived peptides and between (**D**) LptAB of *A. baumannii* with VF16 peptide. ITC experiments were performed in 50 mM sodium phosphate buffer and 150 mM NaCl at pH 7.0. Typically, 25–50 μM proteins in sample cells were titrated with 2 μL of aliquots of peptides, and heat exchange was measured.

We used ITC experiments to determine the interaction parameters of the thanatin analog peptides with LptA_m_ of *E. coli* and LptA homologous protein, LptAB, from *A. baumannii*. Figure 7A–D shows ITC thermograms of the LptA_m_ binding of VM16, VF16, and IM14 peptides. Both VM16 and VF16 demonstrated saturable interactions with LptA_m_, which are exothermic in nature. By contrast, an endothermic binding of IM14 with LptA_m_ is observed with an apparent lack of saturation. Table 6 summarizes the binding and thermodynamic parameters of the interactions. Further, ITC experiments revealed a lack of interactions of VF16 peptide with LptA ortholog of *A. baumannii* (Figure 7). Notably, the VF16 peptide and VM16 did not display antimicrobial activity against the *A. baumannii* strain (Table 1).

## 3. Discussion

Thanatins constitute a family of single disulfide bonded β-sheet host defense peptides that are derived from various species of insects [29,30,31,43]. The spined soldier bug (*P. maculiventris*) thanatin is the first member of the family and is well investigated in terms of structures, activity, and mode of action [29,37,41,42,49,50,51,59]. The N-terminal truncated peptide 16-residue long VM16 and analog VF16 were able to exert growth inhibition of Gram-negative bacteria, including *E. coli*, *K. pneumoniae*, and *S. enterica* (Table 1). Further deletion of the first two amino acids, residues V and P, of VM16 or IM14 have completely abolished the antibacterial activity of the analog (Table 1). These observations largely corroborate with the previous antimicrobial studies of deletion variants of native thanatin [29]. However, the mechanisms of action of these truncated variants were unknown.

**Figure 8:**
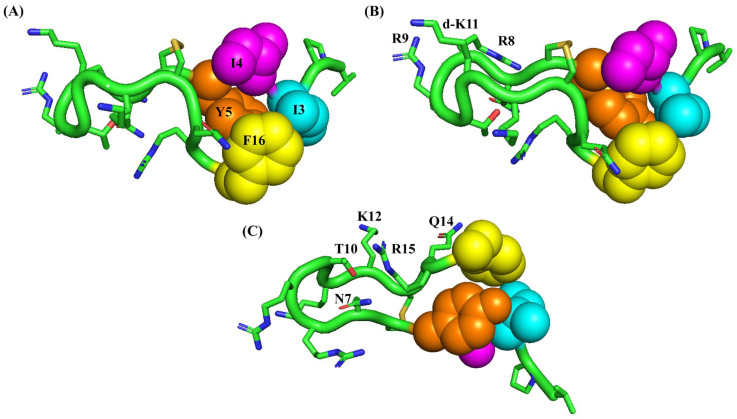
(**A**) The β-hairpin structure of the VF16 peptide determined in complex with LPS micelle depicting close packing interactions among the sidechains of aromatic (Y5 and F16) and aliphatic (I3, I4) residues. (**B**) Proximity of the sidechains of cationic residues, R8, R9, and dk11, in the loop of the β-hairpin structure of VF16 peptide obtained in LPS micelle. (**C**) Disposition of the sidechains of polar and cationic residues N7, T10, Q14, K12, and R15 at the mid-section of the β-hairpin structure of VF16 peptide in complex with LPS micelle.

To correlate with antibacterial activity, we solved atomic-resolution structures of active VF16 and inactive IM14 peptides in free solution and in complexes with LPS-outer membrane. The overall folding of the VF16 peptide, either in free solution or in complex with LPS, is demarcated by the canonical β-hairpin structure. The backbone conformation of the β-hairpin structure of VF16 is better defined in complex with LPS with lower RMSDs of the structural ensembles. The two β-strands are connected by a loop or turn segment consisting of residues N7-R8-R9- T10-dk11. The backbone dihedral angles (ϕ,ψ) of residue dk11 are in α_L_ conformation that can potentially stabilize the orientation of the cationic loop residues. The atomic-resolution structure of VF16 with LPS provides mechanistic insights into the LPS-outer membrane disruption process. As seen, the β-hairpin structure of the VF16 peptide in LPS has rendered significant reorganization of the sidechains of aromatic, aliphatic, and cationic residues. The aromatic sidechains of residues Y5 and F16 and aliphatic sidechains of residues I3 and I4 sustain mutual packing interactions at the termini face of the β-hairpin structure of the VF16 peptide (Figure 8A). The middle and loop regions of the β-hairpin structure describe an extensive cationic and polar surface. The close disposition of the sidechains of residues R8, R9, and dk11 in the loop demonstrates a cationic congregation (Figure 8B). The substitution of residue G11 to dk augments the electrostatic surface of the loop (Figure 5F). The opposite side of the β-hairpin structure of the VF16 peptide delineates a separate cationic-polar face that consists of sidechains of residues N7, T10, K12, and R15 (Figure 8C). These cationic/polar surfaces of the β-hairpin structure of the VF16 peptide could be critical for interactions with negatively charged hydrophilic head groups of the LPS-outer membrane. Whereas the hydrophobic face, a cluster of aromatic-nonpolar sidechains, of the β-hairpin structure might perceive potential non-polar interactions with the acyl chains of the LPS outer membrane. In particular, we surmise that the amphipathic β-sheet structure of VF16 in complex with LPS would efficiently disrupt the outer membrane. The process would essentially permit an efficient translocation of the peptide through OM to the periplasmic space. The VF16 peptide confers a high-affinity binding to LptA_m_, the periplasmic protein component of the LPS transport complex of *E. coli*. It is conceivable that binding of VF16 with LptA_m_ bacterial periplasm would disrupt LPS translocation, inhibiting outer membrane biogenesis. Notably, while our work was ongoing, a recent study employing 16-residue-long thaumatin peptidomimetics demonstrated binding with LptA_m_ protein as a plausible mechanism of Gram-negative bacteria killing following inhibition of outer membrane biosynthesis [50]. High MIC values (>16 μM) estimated for *A. baumannii* can be correlated with the absence of binding of VF16 peptide with LptA ortholog.

The lack of antibacterial activity of the shorter IM14 peptide may be intriguing. A recent study has described the antimicrobial activity and mode of action of a 15-residue N-terminal fragment (PM15) of thanatin [60]. The IM14 peptide demonstrated interactions with LPS and permeabilized the outer membrane like active VM16 or VF16 peptides. However, in ITC studies, the IM14 peptide delineated transient binding with LptA_m_ protein, which would correlate with its lack of antibacterial activity. Atomic-resolution structures of IM14 in free solution and in complex with LPS provide certain insights into interactions with the targets and antibacterial effects. The NMR structure of the free peptide revealed that despite the presence of the disulfide bond, the canonical β-hairpin structure is not maintained for the shorter analog IM14 (Figure 6E). However, the β-hairpin structure of IM14 peptide can be defined in complex with LPS (Figure 6G). In other words, in the complex of the LPS-outer membrane, the β-hairpin structure of the IM14 peptide is sustained, which could be responsible for its ability to disrupt OM permeability. On the other hand, a distorted or flexible β-hairpin structure of IM14 in free solution is unable to confer a high-affinity binding with LptA_m_ protein. We conjecture that IM14 peptide can translocate through the permeabilized LPS-outer membrane into the periplasm space; however, it is unable to inhibit the LPS transport process due to transient binding interactions with LptA_m_. In conclusion, thanatin-based AMPs can serve as a platform for the development of selective and non-toxic potent antibiotics for the treatment of infections caused by MDR Gram-negative pathogens. Structures, activity, and mode of action of thanatin-derived VF16 peptides presented in this work can be exploited for the design of novel antibiotics.

## 4. Materials and Methods

### 4.1. Peptides and Materials

Peptides VM16, VF16, and IM14 were purchased from GL Biochem (Shanghai, China). 1-Nphenylnaphthylamine (NPN) and LPS (*Escherichia coli* O111:B4) were purchased from Sigma Aldrich (Saint Louis, MO, USA). All other chemicals were of analytical grade.

### 4.2. Determination of Minimal Inhibitory Concentration (MIC)

The MIC values of VM16, VF16, and IM14 were estimated against *E. coli* ATCC, *S. enterica* ATCC 14028, *K. pneumoniae* ATCC 13883, *S. pyogenes* ATCC 19615, and *E. faecalis* ATCC 29212, using the broth dilution method. Typically, an overnight bacterial culture was grown to the mid-log phase on a shaker at 183 rpm, at 37 °C, and then diluted in 2X Mueller Hinton (MH) broth (Millipore-Sigma^TM^, Saint Louis, MO, USA) to achieve a final OD of 0.002. In a 96-well plate, 100 μL of the diluted bacterial culture along with 100 μL of peptide, concentrations ranging from 0 μM to 16 μM, was added and incubated overnight at 37 °C. Peptide concentrations were estimated by UV absorbance at a wavelength of 274 nm using a molar extinction coefficient (ε) of 1420 M^−1^ cm^−1^. Bacterial growth inhibition was determined by absorbance of 96 well plates at OD_600_ nm using a Cytation spectrophotometer (Agilent, Santa Clara, CA, USA). The minimum concentration that showed complete inhibition of bacterial growth was determined as the MIC.

### 4.3. Outer Membrane Permeabilization Assay

The outer membrane permeabilization ability of VM16, VF16, and IM14 was determined by performing a NPN uptake assay. Overnight culture of *E. coli* was grown to mid-log phase in LB media and then harvested by centrifugation at 5000 rpm for 10 min. The cells were resuspended in 10 mM sodium phosphate buffer to achieve a final OD_600_ of 0.5. A basal fluorescence of NPN (10 μM) in bacterial suspension, without the addition of peptide, was measured using a Cary Eclipse fluorescence spectrophotometer (Varian, Palo Alto, CA, USA) in a 0.1 cm path-length quartz cuvette. Fluorescence measurements were obtained with excitation set at 350 nm and emission intensities set from 390 to 450 nm. Peptides ranging from 1 μM to 16 µM were gradually added, and maximum fluorescence intensity was then estimated from the emission spectra.

### 4.4. Zeta Potential Measurements

Bacterial surface charge neutralization assay was determined using zeta potential assay. Overnight culture of *E. coli* was grown to mid-log phase in LB media and harvested by centrifugation at 5000 rpm for 10 min, followed by resuspending the cells, OD600~0.01, in 10 mM sodium phosphate buffer, pH 7. The zeta potential of the bacterial cell suspension was obtained in the absence of peptides using zeta cells with gold electrodes. Subsequently, VM16, VF16, and IM14 were added with concentrations ranging from 2 μM to 32 μM. For each concentration, 100 runs were carried out, and three replicates of this assay were performed. All zeta potential measurements were performed on a Zeta Sizer Nano ZS (Malvern Instruments, Worcestershire, UK) equipped with a 633 nm He laser.

### 4.5. MTS Assays of VF16 Peptide with Hek293 and Hep2 Cell Lines

Assays were conducted in complete media for cells Dulbecco’s Modified Eagle Medium (DMEM; Gibco; Waltham, MA USA, catalog number: 11995065) supplemented with 10% fetal bovine serum (FBS; Hyclone; Logan, UT, USA, catalog number: SV30160.03HI-1). VF16 was reconstituted in aqueous solution to a stock of 1 mg/mL. All dose-response peptide dilutions were prepared in a U-bottom 96-well plate. 0.02 × 10^6^ cells/50 µL of complete media were seeded overnight per well in a 96-well plate, and 105 µL of serum-free (sf) DMEM were added. Cells and peptides were incubated at 37 °C/5% CO_2_ overnight. Cell viability was checked using MTS assay and read after 30–45 min, and absorbance at OD490 was read using Tecan Infinite 200 Pro plate reader (Tecan, Mannedrof, Switzerland, CellTiter96^®^AQueous One Solution Cell Proliferation Assay (MTS); catalog number: G3580).

### 4.6. Isothermal Titration Calorimetry (ITC) Studies

The thermodynamic parameters of the thanatin analogs VM16, VF16, and IM14 to LPS and LptA_m_, as well as VF16 to LptA of *A. baumannii* or LptAB were determined by ITC using a Microcal ITC 200 calorimeter (Malvern Panalytical Ltd, Malvern, UK). For binding interactions with LPS, peptides and LPS were diluted in 10 mM sodium phosphate buffer, pH 7.0. 50 µM of LPS was loaded into the sample cell with the same buffer filled in the reference cell, while 1 mM of the peptide was loaded into the syringe. In an individual run, 2.0 μL aliquots of the peptide were titrated into LPS in a sample cell at 37 °C with a stirring speed adjusted to 750 rpm. For LptA_m_ and LptAB binding experiments, LptA_m_, LptAB, and peptides were prepared in 50 mM sodium phosphate buffer, 150 mM NaCl, pH 7.0. 25 mM of LptA_m_ and LptAB were loaded into the sample cell, while 250 mM of VM16, IM14, and VF16 were kept in the syringe. ITC titrations were performed with 20 injections and 7 injections of 2 µL of peptide into sample cells containing LptA_m_ or LptAB, respectively, at 25 °C with a stirring speed of 750 rpm. The raw data was fitted using a single site binding model in Microcal origin 5.0 software to obtain the association constant (Ka) and enthalpy change (ΔH). The dissociation constant (K_d_), free energy (ΔG), and entropy change (TΔS) were calculated as: K_d_ = 1/Ka, ΔG = ΔH-TΔS

### 4.7. Purification of LPS Transport Periplasmic Proteins

Periplasmic proteins involved in LPS transport of *E. coli* and A. baumannii are recombinantly expressed and purified in *E. coli* cells. Recombinant LptA_m_ (residues 28–159) of *E. coli* and LptA (residues 36–182) of A. baumannii were expressed and purified following previous protocols [49]. Plasmids coding for his-tagged proteins were transformed to *E. coli* BL21 cells, and transformants were selected on LB agar plates containing 100 ug/mL of ampicillin incubated at room temperature overnight. Protein expression was induced with 1 mM isopropyl β-d-1-thiogalactopyranoside (IPTG), and the induced bacterial culture was grown at 200 RPM, 18 °C for 18 h [49]. The bacteria cells were harvested by centrifugation at 6000 RPM, 4 °C for 15 min, and resuspended in lysis buffer (100 mM HEPES, 500 mM NaCl, 10 mM imidazole, pH 8). Cells were lysed via sonication on ice at 25 amp for 1 h. The lysate was centrifuged at 18,000 RPM, 4 °C for 30 min to remove cellular debris, and the supernatant collected was incubated with Ni-NTA (Qiagen, Germantown, TN, USA) beads on a rocking platform for 1 h. The column was washed with increasing concentrations of imidazole, and the bound His-tagged proteins were eluted from the beads with elution buffer (20 mM HEPES, 150 mM NaCl, 200 mM imidazole, pH 8). SDS-PAGE was performed for the analysis of eluted fractions. Eluted fractions were concentrated and further purified by size-exclusion chromatography using buffer (50 mM sodium phosphate buffer, 150 mM NaCl, pH, 7).

### 4.8. NMR Studies and Structure Determination of Peptides

NMR spectra were recorded using a Bruker DRX 600 spectrometer equipped with a cryo-probe and pulse field gradients. Two-dimensional ^1^H-^1^H TOCSY (total correlation spectroscopy) and NOESY (nuclear Overhauser effect spectroscopy) spectra of peptides (300 µM) in free solution were acquired with mixing times of 80 ms and 200 ms, respectively. Two-dimensional transferred NOESY (tr-NOESY) experiments were performed, mixing time 150 ms, in the presence of 20 μM LPS (MW of LPS 10 KD). Peptide samples were prepared in an aqueous solution (90% H_2_O, 10% D_2_O), pH 5.0, and NMR data were collected at 278 K. Chemical shift was referenced to DSS (2,2-dimethyl-2-silapentane 5-sulfonate sodium salt) added to the sample. All NMR data were analyzed using TopSpin 2.0 and SPARKY 3 (T.D. Goddard and D.G. Kneller, University of California, San Francisco, CA, USA) software. An ensemble of structures of thanatin peptides VF16 and IM14 were determined using CYANA [61]. Distance restraints were obtained from 2D NOESY or tr-NOESY and were classified as strong, medium, and weak NOEs, which were translated to upper bound distance limits of 2.5 and 3.5. and 5.0 Å, respectively, while the lower distant limits were capped at 2.0 Å. To calculate the structure, a disulfide bond restraint was included between the two cysteine residues. PREDITOR [62] was used to calculate the dihedral angle constraint using Hα chemical shift deviation of individual amino acids. Out of 100 structures calculated, 20 structures with the lowest energy (based on Cyana target function scores) were selected to represent the ensemble and used for further analysis. PROCHECK107 [63] was to validate the quality of structures calculated. Structure figures were generated using PyMOL.

## Figures and Tables

**Table 1:** Minimum inhibitory concentrations (MIC) values (in µM) of VM16, VF16, and IM14 peptides against Gram-negative bacteria; *Escherichia coli* ATCC 25922 (EC), *Klebsiella pneumoniae* ATCC 13883 (KP), *Salmonella enterica* ATCC 14028 (SE), *Acinetobacter baumannii* ATCC BAA-1798 (AB) and Gram-positive bacteria; *Streptococcus pyogen* ATCC 19615 (SP), *Enterococcus faecalis* ATCC 29212 (EF).

Peptides	Gram Negative				Gram Positive	
	EC	KP	SE	AB	SP	EF
**VM16**	2–4	1–2	2	>16	4	4
**VF16**	0.5	1–2	2	>16	1	>16
**IM14**	>16	>16	>16	>16	>16	>16

**Table 2:** Thermodynamic parameters of VM16, VF16, and IM14 peptides binding with LPS in 10 mM sodium phosphate buffer, pH 7.

Peptides	K_d_ (μM)	ΔH (kJ/mol)	TΔS (kJ/mol)	ΔG (kJ/mol)
VM16	1.73	−9.82	24.37	−34.19
VF16	0.30	−18.09	19.61	−37.70
IM14	0.20	−21.31	20.11	−41.42

**Table 3:** Representative long-range NOEs detected for VF16 peptide in free solution and in the presence of LPS micelle.

Free VF16	VF16 in LPS
4 ILE ^α^H–16 PHE H	3 ILE ^δ^H_3_–16 PHE ^δ^Hs
4 ILE ^δ^Hs–14 GLN H	3 ILE ^δ^Hs–16 PHE H
4 ILE ^δ^Hs–15 ARG ^α^H	3 ILE ^δ^H_3_–16 PHE ^δ^Hs
5 TYR H–14 GLN H	4 ILE ^α^H–15 ARG H
5 TYR H–15 ARG ^α^H	4 ILE ^δ^Hs–13 CYS H
5 TYR H–16 PHE H	4 ILE ^δ^Hs–14 GLN H
6 CYS ^α^H–13 CYS ^α^H	4 ILE ^δ^Hs–15 ARG H
6 CYS ^β^Hs–13 CYS ^α^H	5 TYR H–14 GLN H
7 ASN ^β^Hs–12 LYS H	5 TYR ^δ^Hs–14 GLN ^γ^Hs
7 ASN H–12 LYS H	5 TYR ^δ^Hs–15 ARG ^β^Hs
7 ASN ^α^H–13 CYS ^α^H	5 TYR ^δ^Hs–16 PHE ^α^H
	5 TYR ^ε^Hs–16 PHE ^α^H
	6 CYS ^α^H–13 CYS ^α^H
	6 CYS ^β^Hs–13 CYS ^α^H
	6 CYS H–13 CYS ^α^H
	7 ASN ^β^Hs–12 LYS H
	7 ASN ^β^Hs–12 LYS H
	7 ASN H–13 CYS ^α^H
	7 ASN H–14 GLN H
	7 ASN H–15 ARG ^ε^Hs

**Table 4:** Summary of structural statistics of VF16 and IM14 peptides in free solution and in complex with LPS micelle.

	Free VF16	VF16 with LPS	Free IM14	IM14 with LPS
**Distance Constraints**				
Intra-residue [|i − j| = 0]	72	86	62	67
Sequential [|i − j| = 1]	37	45	35	47
Medium range [1 <|i − j| < 4]	8	8	10	9
Long range [|i − j| ≥ 4]	11	20	9	16
Total NOE	128	159	115	136
**Dihedral—angle Constraints (φ, ψ)**	24	24	24	24
**Deviation from mean structure**				
All backbone atoms (Å)	1.02	0.63	1.30	0.79
All heavy atoms (Å)	1.90	1.14	2.37	1.87
**Ramachandran plot for the mean structure**				
% of residues in Most favored region and Additional allowed region	100	100	100	100
% of residues in Generously allowed region	0	0	0	0
% of residues in Disallowed region	0	0	0	0

**Table 5:** Long-range NOEs detected for the IM14 peptide in a free solution and in the presence of LPS.

Free IM14	IM14 in LPS
4 CYS ^α^H–12 GLN H	2 ILE ^δ^Hs–12 GLN ^γ^Hs
4 CYS ^α^H–11 CYS H	2 ILE ^δ^Hs–13 ARG H
4 CYS ^β^Hs–11 CYS H	3 TYR ^δ^H_s_–12 GLN ^β^H_s_
4 CYS ^α^H–11 CYS ^β^Hs	3 TYR ^δ^H_s_–12 GLN ^β^H_s_
5 ASN ^β^Hs–10 LYS H	3 TYR ^ε^H_s_–12 GLN ^β^H_s_
5 ASN ^β^Hs–10 LYS H	3 TYR ^δ^H_s_–13 ARG ^γ^H_s_
5 ASN H–10 LYS H	4 CYS ^α^H–12 GLN H
5 ASN ^γ^Hs–10 LYS H	4 CYS ^α^H–11 CYS H
5 ASN H–11 CYS H^α^	4 CYS ^α^H–11 CYS ^α^H
	4 CYS Hs^β^–11 CYS ^α^H
	4 CYS ^α^H–11 CYS ^β^Hs
	5 ASN ^β^Hs–10 LYS H
	5 ASN ^β^Hs–10 LYS H
	5 ASN H–10 LYS H
	5 ASN ^γ^Hs–10 LYS H
	5 ASN H–11 CYS ^α^H

**Table 6:** Thermodynamic parameters of interactions between VM16 and VF16 peptides with periplasmic protein LptA_m_ of *E. coli*.

Peptides	K_d_ (μM)	ΔH (kcal/mol)	TΔS (kcal/mol)	ΔG (kcal/mol)
**VM16**	0.05	−20.79	−10.82	−9.97
**VF16**	0.25	−8.33	0.68	−9.01

## Data Availability

Data is contained within the article.
